# Traumatic Lumbosacral Dislocation Treated with Posterior Lumbar Interbody Fusion Using Intersomatic Cages

**DOI:** 10.1155/2009/727041

**Published:** 2009-06-16

**Authors:** Katsuhiro Tofuku, Hiroaki Koga, Kazunori Yone, Setsuro Komiya

**Affiliations:** Department of Orthopaedic Surgery, Kagoshima Graduate School of Medical and Dental Sciences, 8-35-1 Sakuragaoka, Kagoshima 890-8520, Japan

## Abstract

A 35-year-old man was struck by a car on his right side and presented with paraparesis of both lower extremities. Radiographic examination revealed multiple transverse process fractures and anterior displacement of L5 on S1. Computed tomography revealed a bilateral anterior facet dislocation of the fifth lumbar vertebra on the sacrum. MRI showed rupture of the posterior ligamentous complex. A posterior lumbar interbody fusion using two intersomatic cages and pedicle screw instrumentation and posterior fusion were performed. Although no major disc lesion was found at the level of L5-S1 on preoperative MRI, a severely collapsed L5-S1 disc was found intraoperatively. Two years after surgery, the patient was asymptomatic with normal neurological findings, and has resumed normal activity. We believe that lumbosacral dislocation can be considered a three-column injury with an L5-S1 disc lesion, and, therefore, requires a solid circumferential segmental arthrodesis to improve fusion rate.

## 1. Introduction

Traumatic lumbosacral dislocation with or without fracture is a rare injury, though it is frequently noted in patients with multiple traumatic injuries [1–8]. This lesion may be unrecognized during initial treatment of patients because of its rarity and the difficulty of examining the lumbosacral junction radiographically. In addition, the optimal treatment of this injury remains controversial. In particular, whether complementary interbody fusion is required in cases without clear evidence of a traumatic L5-S1 disc lesion on preoperative magnetic resonance imaging (MRI) is controversial. We describe a case of traumatic lumbosacral dislocation treated by open reduction and posterior lumbar interbody fusion using intersomatic cages combined with pedicle screw instrumentation and fusion.

## 2. Case Report

**Figure 1:**
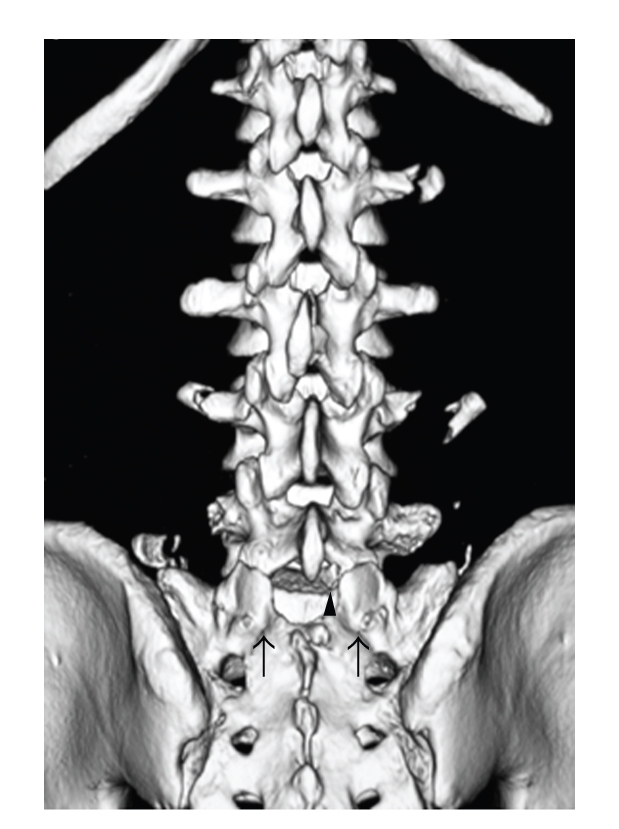
Reconstructed three-dimensional computed tomography demonstrating bilateral anterior facet dislocation with clearly visible sacral articular surfaces (arrows), the fractured tip of the right L5 inferior articular process (arrowhead), and transverse process fractures.

**Figure 2:**
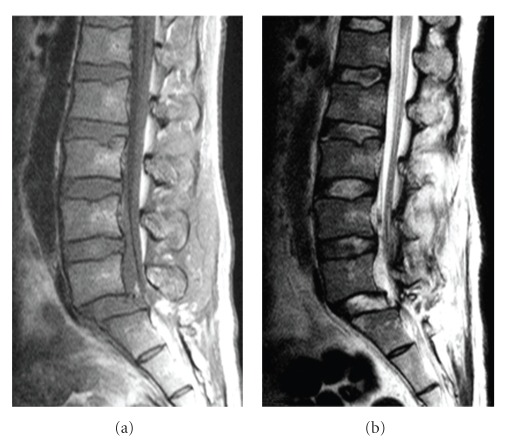
Sagittal magnetic resonance imaging showing anterior displacement of L5 on S1 and disruption of the posterior ligamentous complex. (a) T1-weighted images. (b) T2-weighted images.

A 35-year-old man was struck by a car on his right side while working. He presented with paraparesis of both lower extremities and decreased sensation to light touch and pinprick below the knee bilaterally. He had diminished perirectal sensation and anal sphincter tone. The Achilles reflex was diminished bilaterally. Radiographic examination revealed transverse process fractures bilaterally at L4 and unilaterally at the right L2 and left L5 levels as well as anterior displacement of L5 on S1. Computed tomography revealed a bilateral anterior facet dislocation of the fifth lumbar vertebra on the sacrum with a fracture of the right inferior articular process of L5 and fractures of L2 and L3 spinous processes (Figure 1). MRI showed rupture of the posterior ligamentous complex, but no major disc lesion was found at the level of L5-S1 (Figure 2).

**Figure 3:**
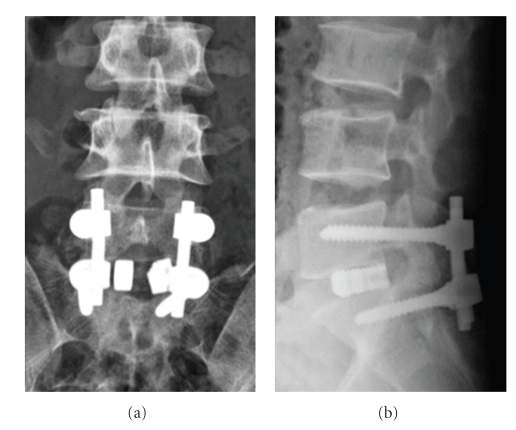
Postoperative anteroposterior (a) and lateral (b) radiographs showing good reduction of L5 anterolisthesis.

 A surgical procedure was performed posteriorly. Ruptured interspinous and yellow ligaments were found at L5-S1 level, and the dural sac was directly visible. In addition, compression of the S1 nerve roots was noted bilaterally. After resection of the ruptured yellow ligament and exploration of the canal at L5-S1 level, complete reduction of the lumbosacral dislocation was achieved without facetectomy by changing the position of the operating table so that the patient's spine exhibited hyperkyphosis and then lordosis relative to the dislocated segment. After reduction, both S1 nerve roots were liberated and decompressed. Posterior lumbar interbody fusion using two intersomatic cages and local bone harvested from the L5 lamina for decompression was performed. Autologous local bone was also used for posterior fusion with segmental L5-S1 pedicle screw fixation. The L5-S1 disc protruded and was easy to resect during the posterior lumbar interbody fusion procedure because it had collapsed severely. Postoperative radiography revealed good reduction (Figure 3).

**Figure 4:**
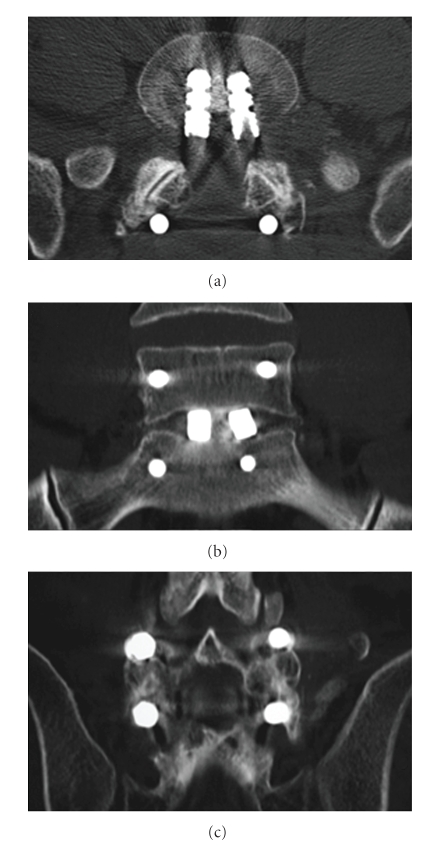
Computed tomography 6 months after surgery demonstrating solid L5-S1 circumferential fusion. (a) Axial section at the lumbosacral junction. (b) Coronal section at the middle portion of the L5 vertebral body. (c) Coronal section at the L5-S1 posterior fusion.

 The patient recovered uneventfully from the operation. Postoperative management consisted of wearing of a lumbosacral corset for 3 months. Six months after the surgery, bony fusion of L5-S1 was observed (Figure 4). At 2 years after the surgery, the patient was asymptomatic with normal neurological findings and has resumed normal activity.

## 3. Discussion

Lumbosacral dislocation is rare, probably because of the frontal-plane orientation of the facets and the intrinsic stability provided by the musculature and iliolumbar ligaments [9]. Most of the cases of this injury previously reported were the result of high-energy trauma and were often associated with other injuries. Transverse process fractures were present in almost all of the reported patients. Although the mechanisms of lumbosacral dislocation are controversial, most authors consider the combination of hyperflexion with compression and rotation the most likely mechanism [1, 2].

 Diagnosis of this lesion requires an initial radiographic study of good quality that demonstrates the abnormal relationships of the lumbosacral facets. However, radiographs taken in the emergency room are often inadequate and can miss this lesion. Patients with severe multiple trauma and fractured transverse processes, which serve as sentinel fractures, should therefore be screened for lumbosacral dislocation. Computed tomography can confirm the diagnosis with axial views of the lumbosacral junction, in which fractures and dislocations can be examined. The “naked facet” sign on axial sections is indicative of facet dislocation. The facets of L5 pass superiorly over those of S1, yielding the appearance of empty or perched facets on computed tomography. Sagittal reconstructions are useful for appreciating the slippage of L5 over S1 and its consequences for the caliber of the vertebral canal. Since injury to the intervertebral disc is a prominent feature of lumbosacral dislocation, MRI should be performed preoperatively to assess the severity of the L5-S1 disc lesion.

 Some authors have reported cases of lumbosacral dislocation nonsurgically treated with satisfactory results [3, 4], although this treatment is not currently accepted since the risk of neurological sequelae is high when performing closed reduction. It is safer to reduce this type of dislocation surgically and essential to explore the vertebral canal at the surgical stage to ensure that there is no potentially neurotoxic bone or disc material before reducing the anterolisthesis of L5 over S1, even if high-quality preoperative MRI is available.

 The use of complementary interbody arthrodesis is controversial. Some authors postulate the importance of interbody arthrodesis only in patients with clear evidence of a traumatic lesion of the disc on preoperative MRI [5, 6]. In addition, a few cases of lumbosacral dislocation without obvious L5-S1 disc lesion on preoperative MRI successfully treated with open reduction and posterolateral fusion with pedicle screw instrumentation alone have been previously reported [5, 7]. In our case, no major disc lesion was found at the level of L5-S1 on preoperative MRI, though the severely collapsed L5-S1 disc was found intraoperatively. We believe that lumbosacral dislocation should be considered a three-column injury with an L5-S1 disc lesion and that solid circumferential segmental arthrodesis is therefore required to enhance fusion rate.

 Pedicle screw instrumentation is the preferred technique for achieving posterolateral fixation. Aihara et al. [10] concluded that posterior lumbar interbody fusion is difficult in lumbosacral dislocation and recommended anterior lumbar interbody fusion after posterior reduction with pedicle screw instrumentation. Verlaan et al. [8] demonstrated that a posterior lumbar interbody fusion procedure using autologous bone graft combined with posterolateral instrumentation and fusion is feasible. However, a posterior lumbar interbody fusion using autologous bone graft alone has the risk of collapse of grafted bone, leading to progressive disc height loss and resulting in malalignment such as kyphosis. In the case of use of intersomatic cages, it is possible to perform a posterior lumbar interbody fusion procedure easily even in cases of lumbosacral dislocation, and to begin rehabilitation early.

 In conclusion, we believe that lumbosacral dislocation can be considered a three-column injury with an L5-S1 disc lesion and therefore requires a solid circumferential segmental arthrodesis to enhance fusion rate. Open reduction and a posterior lumbar interbody fusion using intersomatic cages combined with pedicle screw instrumentation and posterior fusion is reasonable management of this rare injury.
